# The Microsoft HoloLens 2 Provides Accurate Measures of Gait, Turning, and Functional Mobility in Healthy Adults

**DOI:** 10.3390/s22052009

**Published:** 2022-03-04

**Authors:** Mandy Miller Koop, Anson B. Rosenfeldt, Kelsey Owen, Amanda L. Penko, Matthew C. Streicher, Alec Albright, Jay L. Alberts

**Affiliations:** 1 Department of Biomedical Engineering, Cleveland Clinic, 9500 Euclid Ave., Cleveland, OH 44195, USA; koopm@ccf.org (M.M.K.); rosenfa2@ccf.org (A.B.R.); owenk3@ccf.org (K.O.); penkoa@ccf.org (A.L.P.); streicm@ccf.org (M.C.S.); albriga3@ccf.org (A.A.); 2 Center for Neurological Restoration, Cleveland Clinic, 9500 Euclid Ave., Cleveland, OH 44195, USA

**Keywords:** augmented reality, HoloLens 2, validation, gait, turning, timed up-and-go

## Abstract

Augmented-reality (AR) headsets, such as the Microsoft HoloLens 2 (HL2), have the potential to be the next generation of wearable technology as they provide interactive digital stimuli in the context of ecologically-valid daily activities while containing inertial measurement units (IMUs) to objectively quantify the movements of the user. A necessary precursor to the widespread utilization of the HL2 in the fields of movement science and rehabilitation is the rigorous validation of its capacity to generate biomechanical outcomes comparable to gold standard outcomes. This project sought to determine equivalency of kinematic outcomes characterizing lower-extremity function derived from the HL2 and three-dimensional (3D) motion capture systems (MoCap). Sixty-six healthy adults completed two lower-extremity tasks while kinematic data were collected from the HL2 and MoCap: (1) continuous walking and (2) timed up-and-go (TUG). For all the continuous walking metrics (cumulative distance, time, number of steps, step and stride length, and velocity), equivalence testing indicated that the HL2 and MoCap were statistically equivalent (error ≤ 5%). The TUG metrics, including turn duration and turn velocity, were also statistically equivalent between the two systems. The accurate quantification of gait and turning using a wearable such as the HL2 provides initial evidence for its use as a platform for the development and delivery of gait and mobility assessments, including the in-person and remote delivery of highly salient digital movement assessments and rehabilitation protocols.

## 1. Introduction

Identifying individuals at risk of falling remains a critical unmet need across neurological populations and older adults [1,2]. Gait speed has been proposed to be the “the sixth vital sign” [3], as it has been shown to predict hospitalizations, rehabilitation destinations, and falls [4,5,6,7]. Similarly, the Timed Up and Go Test (TUG) is one of the most widely used clinical tests of functional mobility in neurological and geriatric populations [8], and provides an assessment of walking, turning, and postural transitions, including standing and sitting [9,10]. Similar to gait speed, the TUG is a reliable and valid measure of fall prediction in older adults [11]. While there is an abundance of data characterizing gait and TUG metrics in older populations and in those with neurological disease, the assessment of gait speed and functional mobility in a clinical setting is inconsistent. The fields of rehabilitation and neurology continue to identify a solution to provide a user-friendly, objective assessment of functional mobility that can be integrated into busy clinical workflows [12,13,14].

Augmented reality (AR) may be a technology solution for the routine delivery and assessment of gait and functional mobility metrics in clinical settings. Head-mounted AR systems deliver an interactive experience to the user by placing digital objects in the user’s real-world environment [15]. Notably, AR systems are untethered wearables with an embedded inertial measurement unit (IMU) and a depth sensor that can capture kinematic data to quantify the user’s activities (e.g., sitting, standing, walking, and turning). Because AR systems have the potential to deliver movement cues and objectively quantify movement, they are candidates for integration into clinical workflows for the delivery of movement assessments. However, AR motion sensors must be rigorously vetted to establish the validity and reliability of the data prior to clinical integration. Recently, the first generation of head-mounted AR system, HoloLens 1 (HL1) (Microsoft Corporation, Redmond, CA) was validated for the assessment of gait and physical mobility [16,17]. Overall, the studies revealed good agreement between the HL1 outcomes for measures of accuracy in step detection and overall task time. However, other key metrics for characterizing locomotion were not evaluated for accuracy due to significant errors in the reference motion sensors [16], and known-group validity analyses provide inconsistent results [16,17].

We recently completed a validation study of the HL1 motion sensors to characterize gait compared to a 3-dimensional (3D) motion capture (MoCap) system [18]. The results indicated that the 3D position and orientation data from the HL1 resulted in the accurate and reliable calculation of spatiotemporal gait variables (≤5% error) during straight-line walking compared to a MoCap system across multiple walking speeds [18]. The preliminary results were very promising. Since the initial validation study, Microsoft Corporation released the HL2 to address the hardware and software limitations of the HL1, including its restricted field of view, limited battery life, and relatively heavy headset, which limited its wear time.

The subsequent HL2, with an enhanced field of view (52 degrees), reduced weight (566 g), and improved battery life (3 h), was released in November 2019 and is commercially available. The systematic validation of the HL2 motion sensors is a necessary precursor to using metrics derived from the HL2 in clinical applications or in the study of gait deficiencies in neurological populations. The primary aim of this project was to determine whether the data derived from the HL2 characterizing lower extremity function during continuous walking and the TUG were equivalent to the outcomes derived using the gold standard MoCap system. Based on positive pilot studies using the HL1 [16,17,18,19], it was hypothesized that the biomechanical outcomes of gait, turning, and functional mobility from the two systems would be equivalent.

## 2. Methods

### 2.1. Participants

Sixty-six participants were recruited with flyers from the Cleveland metropolitan area and participated in this validation study from May 2020 to June 2020. All participants completed the informed consent process approved by the Cleveland Clinic IRB. Inclusion criteria were as follows: age 18–45 years and the self-reported ability to ambulate 20 min continuously. Exclusion criteria included: cardiovascular, musculoskeletal, metabolic, or neurological disease that impairs one’s ability to ambulate unassisted, history of concussion in the previous six months, or a history of concussion at any time with residual physical or cognitive symptoms, and the use of an assistive device during standing or ambulation. The age group was selected due to the relative homogeneity in gait speed, variability, and spatiotemporal variables among the men and women [20,21].

### 2.2. Procedures

Participants completed a single data collection session within the Biomechanics Laboratory at the Cleveland Clinic. During the visit, participants performed two experimental tasks while kinematics data were collected concurrently with the MoCap and HL2 systems: (I) 60 s of continuous walking along an oval path (7 × 2 m walkway) at their preferred speed, and (II) TUG. An oval path was selected for the walking assessments to allow a continuous walking trial, which has been shown to produce gait speeds that more accurately represent normal walking speed compared to discrete walking trials [22]. A straight-line segment (4 × 2 m) of the oval path (7 × 2 m walkway) was positioned within the MoCap capture volume (4 × 2 m). Following instruction, the participants first verified that they understood the tasks and that the equipment did not impede their normal movements; they then completed a single trial for each task. Trials were initiated by an audible 5 s countdown delivered to the participant through the HL2 headset. After the completion of each trial, the testing administrator performed visual assessments of the data to ensure data were recorded and files were named appropriately.

### 2.3. Equipment, Processing, and Data Analysis

**Figure 1:**
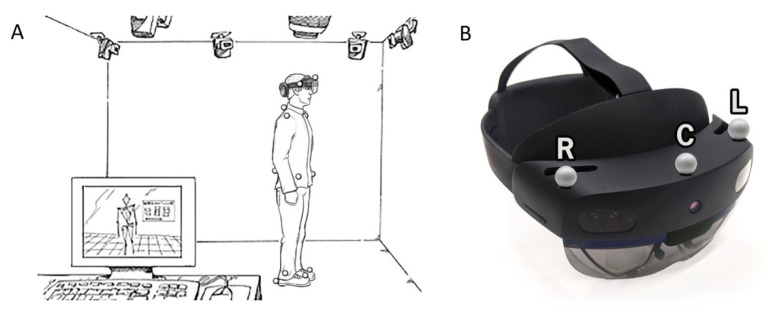
(**A**) Experimental set-up. An eight-camera MoCap system was used as the gold standard for movement analysis. Twelve retroreflective markers were placed on anatomical landmarks; (**B**) three additional markers (R—right, C—center, and L—left) were placed on the HL2 headset.

Three-dimensional position data gathered from an eight-camera MoCap system (Motion Analysis Corp., Santa Rosa, CA, USA) served as the gold-standard system [23]. During the walking and TUG tasks, 3-D position data from 15 retroreflective markers were utilized. Twelve markers were placed on the following anatomical locations for data collection with the MoCap system: bilateral acromion, the 7th cervical vertebra, bilateral anterior superior iliac spine, the sacrum, bilateral 2nd metatarsal, bilateral lateral malleolus, and bilateral heel. Furthermore, three markers were placed on the left, right, and center portions of the HL2 visor (Figure 1). Data were sampled from the MoCap system at 60 Hz.

The HL2 is an AR headset with four visible light cameras, two infrared cameras, a depth sensor, and an IMU. The 3D position and 3D rotation data of the headset were sampled at approximately 60 Hz; a linear interpolation was used to ensure a constant sampling frequency of 60 Hz to match the MoCap system.

Motion data from both systems were filtered with a fourth-order low-pass Butterworth filter with a cutoff frequency of 2 and 2.5 Hz for gait and TUG trials, respectively. A synchronization computer sent a pulse to a host Raspberry Pi to signal the initiation of data collection for both systems, which were initiated within 10–100 ms of one another. An orthogonal correction was applied utilizing a rotation of the HL2 data about the vertical axis to spatially align the two coordinate systems. Motion data for when an individual ambulated outside the capture volume of the MoCap system were removed from the HL2 dataset. Additional details regarding data synchronization and alignment are provided in our previous publication [18].

### 2.4. Continuous Gait Biomechanical Variables

Following data alignment, 3D positional data from MoCap markers (right heel, left heel, and markers on the HL2 headset) and 3D positional and rotational data of the headset from the HL2 were used to calculate biomechanical metrics of gait, which included: cumulative distance, number of steps, step and stride length, total time, and gait velocity. Details of the mathematical calculations have been published previously [18].

### 2.5. Timed Up-and-Go Biomechanical Variables

**Figure 2:**
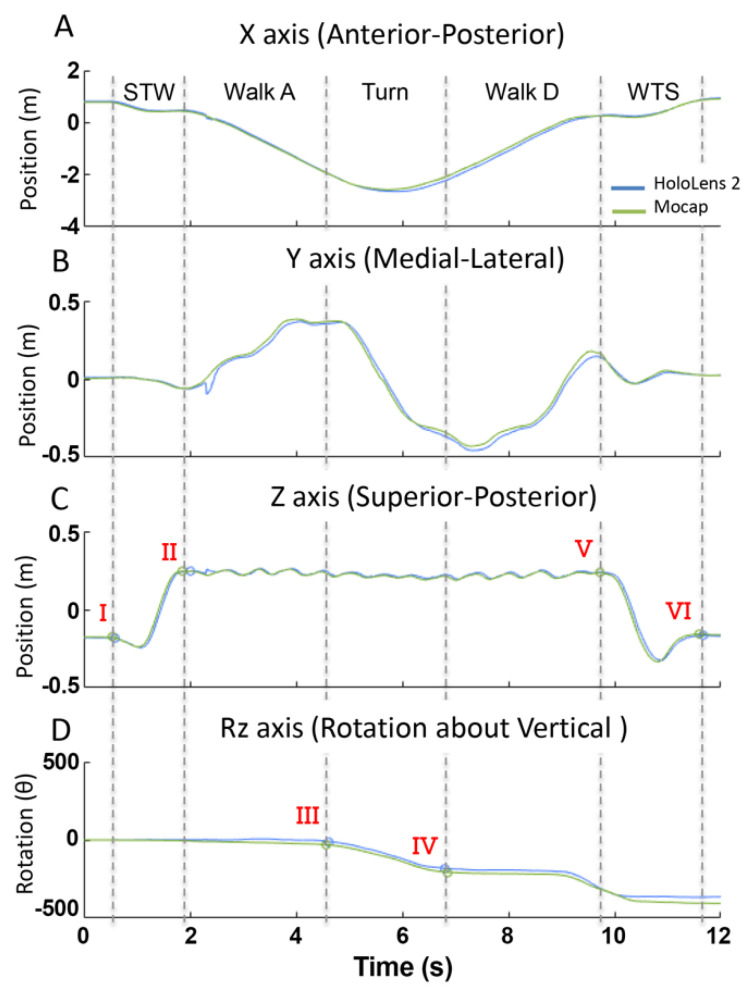
Position (**A**–**C**) and rotation (**D**) data from the MoCap (green) and HL2 (blue) from a representative TUG trial. The five phases of the TUG were segmented (dashed vertical lines) using the vertical position data (**A**) and vertical rotation data (**D**) and included: sit-to-walk (STW), gait ascend (Gait-A), turn, gait descend (Gait-D), and walk-to-sit (WTS). Points I–V are time points used in the algorithm to segment the trial.

Temporal and biomechanical metrics from the HL2 and MoCap systems for the TUG task were calculated independently. For each system, data were used to segment the trial into the five phases of the TUG: sit-to-walk, gait ascend, turn, gait descend, and walk-to-sit. Specifically, vertical position data were differentiated to calculate the vertical velocity data. Zero-crossings in the velocity data were used to identify the initiation and termination of the sit-to-walk phase (Figure 2C, point I-II, respectively), the initiation of the walk-to-sit (Figure 2C, point-V), and the end of the trial (Figure 2C, point-VI).

The turning and gait phases were segmented using a previously validated algorithm for turn detection [10,24]. Specifically, the onset and offset of turning were determined using a theoretical model of straight-line walking and 180° turning motion that was fitted to the vertical rotation data (Rz) using a least-squares optimization algorithm. For the MoCap system, angular rotation of the headset about the vertical axis (Rz) was calculated using the 3D position data of the retroreflective markers secured to the HL2 headset (Figure 1B). To calculate Rz, a 2D unit vector in the horizontal plane from the center to the left MoCap markers secured to the HL2 headset (Figure 1B) was calculated for each time sample. The angle between the unit vector at time sample i and time sample i + 1, where i = 1:total number of samples, was calculated using the dot product [25]. Once the angular position was calculated for the MoCap system, a mathematical model for 180-degree turns, based on simple angular position, was utilized to detect turn onset and offset. The mathematical model fitted a line with three segments: a horizontal line, a line segment with constant slope, and a second horizontal line [24]. The transition points from zero slope to constant slope marked the onset (Figure 2D, point III) and offset (Figure 2D, point IV) of the turns [24]. Turn duration (s) was calculated using the turn onset and offset time points. Gait ascend and descend duration phases (s) were segmented automatically through the aforementioned analyses, and metrics from these two phases were combined to create one set of gait metrics per trial. Gait duration (s) was the total time spent walking and Gait velocity (m/s) was calculated as the total distance traveled in the horizontal plane (m) divided by the Gait duration (s). The number of steps (count) and step length (m) were defined using the same algorithm from the continuous walking data, and calculated during the gait and turning phases, respectively. Turn velocity (deg/s) was calculated as the rotational displacement about the vertical axis measured during the turning phase divided by the Turn duration. Peak turn velocity (deg/s) was calculated as the maximum rotational displacement between successive samples and the time increment between two samples.

For the HL2 metrics, the same algorithm was applied for segmenting the TUG trial into the five phases. Unlike the MoCap system, the angular rotation (Rz) of the headset about the vertical axis was a direct output from the HL2 system and, therefore, did not require calculation.

### 2.6. Statistical Analysis

Criterion validity for the biomechanical outcome metrics calculated using raw data from the HL2 system and the MoCap system was determined by Bland–Altman (BA) analyses and two one-sided paired-t-tests for equivalence testing (TOST) [26,27]. For each metric, the a priori equivalence margin for the TOST tests was set to +/−5% of the mean MoCap measurement [28,29,30]. The reliability of the HL2 metrics was evaluated using the intra-class correlation coefficients (ICC(2,1)) based on two-way random effects models for single-rater consistency with 95% confidence intervals. ICC values were classified according to the following guidelines: values <0.5 indicated poor reliability, values between 0.5 and 0.75 indicated moderate reliability, 0.75–0.9 indicated good reliability, and greater than 0.90 indicated excellent reliability [31].

The RMS errors were calculated in the x, y, and z axes, as well as the angular rotation about the vertical axis (Rz) in the TUG tasks in order to assess alignment in the time series data between the HL2 and MoCap.

## 3. Results

### 3.1. Participant Demographics

Sixty-six participants completed the continuous walking and TUG tasks. MoCap data from two participants were missing due to marker occlusion. Therefore, all the outcomes were calculated from the remaining data from 64 participants (Table 1).

### 3.2. Time-Series Data Demonstrate Excellent Agreement for Continuous Walking

The alignment of the time series position data between the MoCap and HL2 systems was calculated using the root-mean-squared-error (RMS error) for each axis (X, Y, and Z). The time series data of the HL2 showed excellent agreement with the MoCap data based on RMS error values of 8.5 cm, 2.1 cm, and 0.8 cm in the X, Y, and Z axes, respectively.

### 3.3. Biomechanical Outcomes across Systems Are Equivalent during Continuous Walking

**Figure 3:**
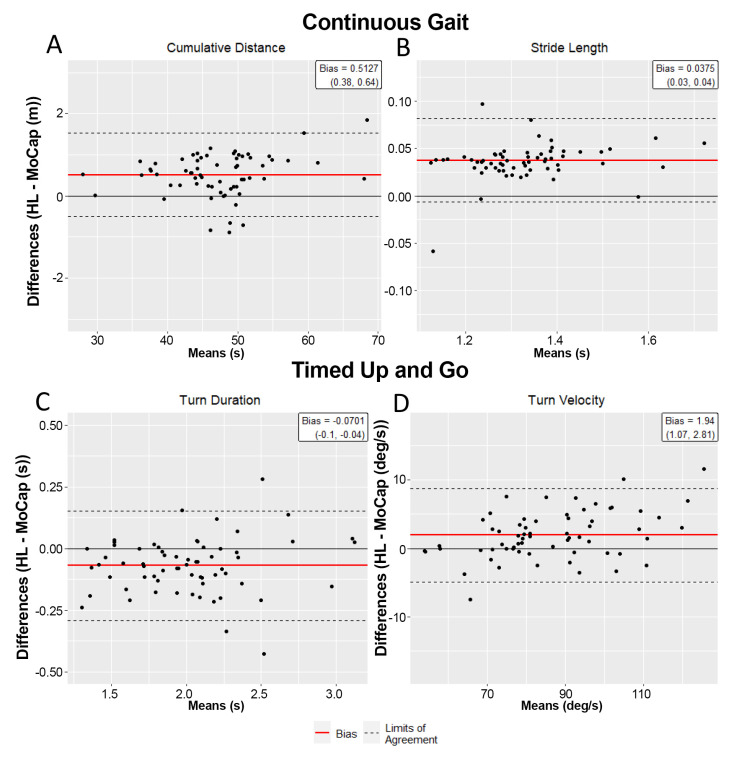
Bland–Altman plots for continuous walking (**A**,**B**) and TUG (**C**,**D**) metrics. (**A**) Average cumulative distances over the 60 s walk for the HL2 and MoCap were 47.46 (7.26) and 46.95 (7.15) m, respectively, with the average bias (HL2-MoCap) of 0.51 m and LOA of (−0.50, 1.52) m. (**B**) For average stride length, the average bias was 0.04 m with LOA of (−0.01, 0.08) m. (**C**) For turn duration, the average bias of the HL2 was −0.07 s with LOA of (−0.29, 0.15) s. (**D**) For turn velocity, the average bias was 1.94 deg/sec with LOA of (−4.89, 8.77) deg/s.

Figure 3 depicts the BA plots for the cumulative distance and stride length variables. On average, the HL2 was within 1.1% and 3.1% of the MoCap system in determining the cumulative distance and stride length measures, respectively. The average difference between each data pair, the bias (HL2–MoCap), is included in Table 2 for all the continuous walking outcomes. The TOSTs determined that across all the gait outcomes, the HL2 measures were statistically equivalent (error ≤ 5%, *p* < 0.05) to the MoCap measures at a 95% confidence level (Table 2). Correspondingly, the reliability of the HL2 metrics for the continuous walking trials were found to be excellent, with all the ICC (2,1) values ≥0.98.

### 3.4. Time Series Data Demonstrate Excellent Alignment for TUG Task

The 3D positional and rotational data from a representative TUG trial are shown in Figure 2. Across all axes, the kinematic data from the HL2 motion sensors demonstrate excellent temporal and spatial agreement with the MoCap measures. The RMS errors between the two systems for the given trial were 4.8, 2.1, and 1.2 cm in the x-, y-, and z-axes, respectively, and 1.5 deg in the vertical rotation data.

Across all the participants, the alignment of the time series position and vertical rotation data of the HL2 and MoCap showed excellent alignment based on average RMS error values of 8.5 cm, 3.2 cm, and 2.4 cm in the x-, y-, and z-axes, respectively, and 9.0 deg in the vertical rotation data (Figure 2).

### 3.5. HL2 Is Eequivalent to Motion Capture at Characterizing TUG

The phases of the TUG were parsed using the algorithm described in the Methods section. Figure 3 depicts the BA plots for the primary turning measures derived from the HL2, turn duration and mean turn velocity, which were within 3.5% and 2.2% of the MoCap measures, respectively. A total of twelve outcome metrics was calculated to characterize the TUG performance for each system and the results of the equivalence testing are provided in Table 3. Notably, nine of the 12 TUG outcomes were statistically equivalent between the MoCap and HL2 (error ≤ 5%; *p* < 0.05 for all TOSTs tests). The sit-to-walk duration, walk-to-sit duration, and peak turn velocity were not statistically equivalent between the two systems, although the absolute percentage difference for each measure was less than 5%. The ICC values for all the TUG metrics were ≥0.79, indicating good-to-excellent reliability between the systems.

## 4. Discussion

The results from this project support the hypothesis that biomechanical outcomes characterizing lower extremity function during walking and functional gait, derived from the HL2 and gold standard motion capture, are equivalent to one another. Equivalency between systems support and expand our earlier work, indicating that first-generation HL1 data could be used to evaluate walking patterns [18]. Notably, the participants in the current study walked a substantially greater distance compared to our earlier project (47 m versus 3 m). Evaluating the accuracy of the HL2 at characterizing gait over a greater distance was a critical unanswered question, as one of the appealing features of an AR system is that it is untethered, thereby allowing the precise quantification of unconstrained, potentially “real-world” movement. The equivalence across the systems in terms of walking distance indicates the HL2 measurements and data collection hardware are relatively stable and reliable; there was negligible drift between the two systems, as the participants walked nearly 50 m. Further, the biomechanical outcomes such as step length, stride length, and walking velocity were equivalent between the systems. The ability to accurately quantify biomechanical measures of gait over relatively long distances expands the potential to use AR technology in populations living with different neurological diseases, such as Parkinson’s disease (PD) and multiple sclerosis, whose gait patterns may worsen with increasing distance [32,33].

The results of this study demonstrate that the HL2 can be used to objectively and reliably quantify functional gait assessments, such as the TUG, which provides the cornerstone for potential clinical use. Importantly, the data from the HL2 provided sufficient resolution to parse the TUG into meaningful components by providing granular biomechanical measures of movement patterns compared to the traditional, somewhat generic, total time to complete. Providing a user with detailed biomechanical outcomes that precisely characterize functional mobility has the potential to facilitate integration into clinical use. Typically, the TUG has been used to assess overall mobility in geriatric and patient populations [11]. A recent normative study of healthy adults (20–60 yrs.), reported differences in total trial time for older adults (50–60 yrs.) compared to those in their 20 s, 30 s, and 40 s, but not between the younger age groups [34]. Significant differences in TUG times were related to reduced socioeconomic status, increased body mass index, and comorbidities. These results provide insight into the contributing factors that reduce performance for older adults, but further work is required to identify the specific impairments that result in longer trial times, and whether significant differences in biomechanical measures exist between younger age groups. Given the demonstrated accuracy of the HL2, future normative studies of healthy adults utilizing the HL2 device could evaluate biomechanical measures (e.g., gait or turning velocity) to determine specific impairments and identify physical therapy interventions tailored to improve movement performance as it deteriorates with age.

The identification and quantification of turning using the HL2 address important and meaningful gaps in the assessment of functional mobility in neurological patient populations. Turning is a complex motor task that is frequently associated with fall risk in healthy older adults [35] and individuals with PD [36]. Difficulties in turning, such as low turning velocity and increased step frequency, are particularly problematic in individuals with PD and can lead to the freezing of gait and falls [37]. Previous studies have shown that falls that occur while turning led to an eight-fold increase in hip fractures compared to straight-path falls [38,39,40], and medical costs more than tripled when a person sustained a broken hip from a fall [41]. The importance of treating lower-extremity dysfunction in PD, such as the freezing of gait, is well recognized. However, triggering these symptoms during a clinical examination or physical therapy sessions remains challenging [42,43], partly due to the difficulty in replicating real-world environments in a clinical setting that are known to trigger FOG. AR systems, like the HL2, contain the necessary functionality to bridge the gap in clinical assessments of fall risk by providing a platform that can deliver an interactive digital environment to replicate the context in which instrumental daily activities that represent a fall risk are completed, while simultaneously providing valid and precise outcome measures.

The HL system has been examined as a rehabilitation tool in the clinical arena and utilized to provide digital content to individuals with PD, with the aim of improving spatio-temporal measures of gait, turning, and improvements in balance stability [44,45,46,47]. These studies demonstrate the feasibility of using AR systems, such as the HL2, to deliver a therapeutic intervention in patient populations; however, the researchers did not utilize the HL motion sensors to measure performance. Rather, they relied on an external motion capture system or clinical scales to quantify performance. The validation of the derived biomechanical measures of gait, turning, and functional mobility from the HL2 motion sensors, as demonstrated in the current study, suggests that systems external to the HL2 may not be necessary as it provides data that are sufficiently accurate to characterize common aspects of lower-extremity function. Subsequent studies involving AR technology, such as the HL2, can now be performed utilizing the full capability of the systems to deliver digital content that the user can interact with, such as stepping over a digital curb, while providing trusted validated measures of lower-extremity function.

Taken together, the results of this project demonstrate that the HL2 provides standardized and accurate data for the determination of objective measures of functional gait that could improve clinical decision-making and the care of patients with lower extremity deficits. The concept of integrating wearable sensors to evaluate human movement using novel approaches is gaining momentum [48]. Tetherless AR technology has the potential to differentiate itself from other portable, inexpensive IMU-based systems, as they can enhance users’ environment with holographic images to add cognitive and physical complexity, which can reliably provoke symptoms and produce biomechanical measures of movement performance through the device’s embedded motion sensors. The barriers associated with the use of the AR systems for quantitative measures of functional gait in the clinical context are relatively limited as it is a stand-alone system that includes a single component (AR headset) compared to multiple IMUs or a multi-marker, multi-camera, motion analysis system. Consistent with the adoption of any new technology in medicine, clinician time and willingness to adapt are likely barriers to its integration into clinical workflows. However, we have demonstrated that the integration of technology into clinical workflows, when properly developed and validated, can improve outcomes and reduce cost [49], as well as decreasing the time physicians spend documenting in the electronic health record [50]. While this project focused on the validation of the HL2 device, other head-mounted AR systems are available. Alternative systems, such as the Magic Leap One and Magic Leap Two, vary slightly in hardware design and user interface, provide a slightly larger field of view, and are marginally less expensive, at $2.5K/unit for Magic Leap One and $3.5K/unit for HL2. However, to our knowledge, no other commercially available AR system has undergone the rigorous validation process of the HL2. The validation of other AR systems is necessary, as each contains a different IMU package, with varying measurement resolutions.

There are limitations to this project. The participants were healthy and young-to-middle-aged. Given the well-documented changes in gait that are part of the normal aging process, validation on a group of older adults is warranted. Furthermore, validation should also be considered in individuals with atypical gait patterns. A recent study validated the spatiotemporal gait parameters calculated from the HoloLens 1 with a non-gold-standard motion system (interactive walkway) in a group of individuals with PD and young healthy adults [17]. The results demonstrated a significant difference between young and PD and excellent test–retest reliability and between-systems agreement (intraclass correlation coefficient >0.80) for gait metrics (gait speed, step length, and cadence). These preliminary results in a PD patient group are promising; however, the disease severity of the patient cohort, Hohen and Yahr stage 2.2, typically involves minimal gait impairments [51]. Thus, further research is required to determine whether the HL2 can provide biomechanical measures of functional gait, including step count and turning, for patient groups with impaired movements, such as shuffle-gait and the freezing of gait, which is often seen in moderate–advanced-stage PD. Finally, since the postural transfers (sit-to-walk and walk-to-sit) in our dataset did not reach statistical significance, further work validating the HL2 with movements in the vertical axis, such as squatting or kneeling, is necessary.

As AR technology continues to evolve, applications that involve the delivery of interactive mobility evaluation and treatment protocols with simultaneous movement assessment should be evaluated. Specifically, our team is pursuing the utilization of AR technology to deliver a dual-task intervention to treat postural and gait deficits in individuals with PD and to assess mild traumatic brain injury. The systematic and rigorous evaluation of the measurement capabilities of AR technology is the prerequisite for clinical integration; the results from this project suggest AR technology is viable to provide value in the evaluation of neurological patients. 

## 5. Conclusions

The biomechanical outcomes characterizing lower-extremity function during walking and functional gait, derived from the HL2 and gold standard motion capture, were statistically equivalent to one another for the majority of outcomes. The accurate quantification of gait and turning using the HL2 provides initial evidence for its use as a platform for the development and delivery of gait and mobility assessments, including the in-person and remote delivery of rehabilitation protocols.

## Figures and Tables

**Table 1:** Participant demographics (*n* = 64).

Age 18–44 (Years) Cohort by Age Group	26.8 (6.4)
18–23	23 (35.9%)
24–40	38 (59.4%)
40–44	3 (4.7%)
Height (in)	68.4 (3.9)
Weight (lbs.)	164.9 (41)
BMI	24.6 (4.4)
Gender	
Female	29 (45.3%)
Male	34 (53.1%)
Other/No Response	1 (1.6%)
Race	
Asian	2 (3.1%)
Black or African American	0 (0%)
White	60 (93.8%)
More than One Race	1 (1.6%)
Unknown/Not Reported	1 (1.6%)
Years of Education	16.5 (2.2)

Note: Overall age, height, weight, years of education, and BMI expressed as mean (SD); all others expressed as count (%).

**Table 2:** Biomechanical outcomes from the MoCap and HL2 systems for continuous walking task.

	MoCap Mean (SD)	HoloLens 2 Mean (SD)	Bias (95% CI)	ICC (95% CI)
Cumulative distance (m)	46.95 (7.15)	47.46 (7.26)	0.51 (0.38, 0.64) *	0.99(0.99, 0.99)
Number of steps (count)	51.39 (5.70)	51.53 (5.61)	0.14 (−0.04, 0.32) *	0.99(0.99, 1.00)
Step length (m)	0.64 (0.06)	0.66 (0.06)	0.02 (0.02, 0.02) *	0.99(0.99, 1.00)
Stride length (m)	1.31 (0.12)	1.35 (0.12)	0.04 (0.03, 0.04) *	0.98(0.97, 0.99)
Total walking time (s)	29.74 (1.75)	29.72 (1.74)	−0.02 (−0.03, 0) *	0.99(0.99, 0.99)
Gait velocity (m/s)	1.59 (0.23)	1.61 (0.24)	0.02 (0.02, 0.03) *	0.99(0.99, 0.99)

* = TOST *p*-Value < 0.05; bias is defined as (HL2 measure–MoCap measure).

**Table 3:** Biomechanical outcomes from the MoCap and HL2 systems for timed up-and-go task.

	MoCap Mean (SD)	HoloLens 2 Mean (SD)	Bias (95% CI)	ICC (95% CI)
Total trial duration (s)	10.82 (1.59)	10.7 (1.56)	−0.12 (−0.2, −0.03) *	0.98 (0.96,0.99)
Sit-to-walk duration (s)	1.56 (0.37)	1.52 (0.33)	−0.04 (−0.09, 0.01)	0.84 (0.76,0.90)
Walking time (s)	6.79 (1.17)	6.84 (1.11)	0.05 (0, 0.1) *	0.98 (0.98,0.99)
Turn duration (s)	2.04 (0.41)	1.97 (0.42)	−0.07 (−0.1, −0.04) *	0.96 (0.94,0.98)
Walk-to-sit duration (s)	2.47 (0.67)	2.35 (0.6)	−0.12 (−0.21, −0.03)	0.85 (0.76,0.91)
Turn velocity (deg/s)	85.24 (15.79)	87.18 (17.25)	1.94 (1.07, 2.81) *	0.99 (0.99,1.00)
Peak turn velocity (deg/s)	125.99 (23.02)	131.1 (30.62)	5.1 (0.74, 9.47)	0.79 (0.68,0.87)
Gait velocity (m/s)	0.94 (0.11)	0.97 (0.11)	0.03 (0.03, 0.04) *	0.99 (0.99,1.00)
Number of steps in a turn	3.23 (1)	3.23 (0.94)	0 (−0.15, 0.15) *	0.82 (0.71,0.88)
Number of steps	10.89 (1.76)	11.2 (1.65)	0.31 (0.14, 0.48) *	0.92 (0.87,0.95)
Step length (m)	0.59 (0.07)	0.6 (0.06)	0.01 (0, 0.02) *	0.87 (0.79,0.92)
Cumulative distance (m)	8.09 (0.46)	8.18 (0.44)	0.09 (0.07, 0.12) *	0.98 (0.96,0.98)

* = TOST *p*-Value < 0.05; bias is defined as (HL2 measure–MoCap measure).

## Data Availability

The data presented in this study are available on request from the corresponding author.
